# Supplementary material for: Integration of Mutational Signature Analysis with 3D Chromatin Data Unveils Differential AID-Related Mutagenesis in Indolent Lymphomas
Source: Int J Mol Sci. 2021 Dec 1;22(23):13015. doi: 10.3390/ijms222313015 (PMC8657711; doi:10.3390/ijms222313015)

Supplementary Figures

Figure S1 Evaluation of the optimal signatures number.

Figure S2 Cluster silhouette analysis for divisive hierarchical clustering.

Figure S3 Mutational load and distribution of variant allele frequency in FL and CLL mutated (CLL M) and unmutated (CLL U).

Figure S4 96-trinucleotide mutational pattern of FL and CLL/MBL.

Figure S5 Characterization of immunoglobulin loci (IG) in FL and CLL samples.

Figure S6 Consistency of signature extraction with sample bootstrapping.

Figure S7 De novo mutational signatures extraction in immunoglobulin genes loci (IG).

Figure S8 Heatmap of DNA repair genes mutated in FL and CLL/MBL.

Supplementary Figure 1. Evaluation of the optimal signatures number. We calculated the residual signature average stability (average silhouette coefficient) and the mean sample cosine distance to define to optimal signatures number.

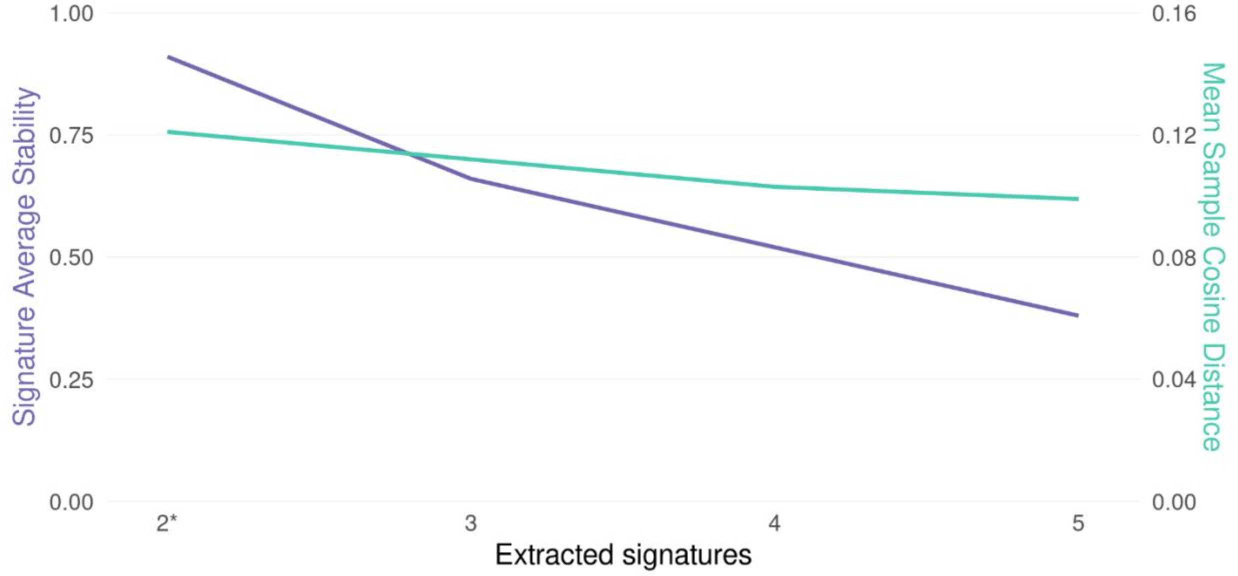

Supplementary Figure 2. Cluster silhouette analysis for divisive hierarchical clustering. Silhouette coefficient will range for each sample from -1 (if it is wrongly placed on the current cluster) to 1 (if it is correctly placed).

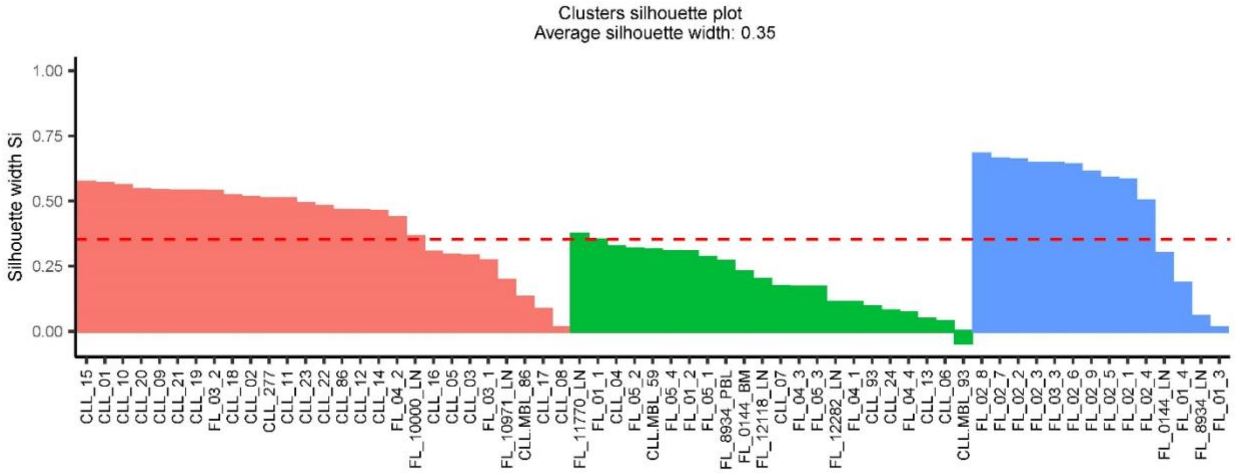

Supplementary Figure 3. Mutational load and distribution of variant allele frequency in FL and CLL mutated (CLL M) and unmutated (CLL U). **A** The mutation rate expressed as the number of mutations per exome was higher in FL (*n*=33) than in CLL M/CLL U(*n*=30) (t-test, two-sided). **B** Violin plots depict a distinct distribution of variant allele frequency in FL and CLL M/CLL UM. White circle: Median variant allele frequency; Colored bars: 25th and 75th percentiles; Whiskers: 5th and 95th percentiles (Wilcoxon test.)

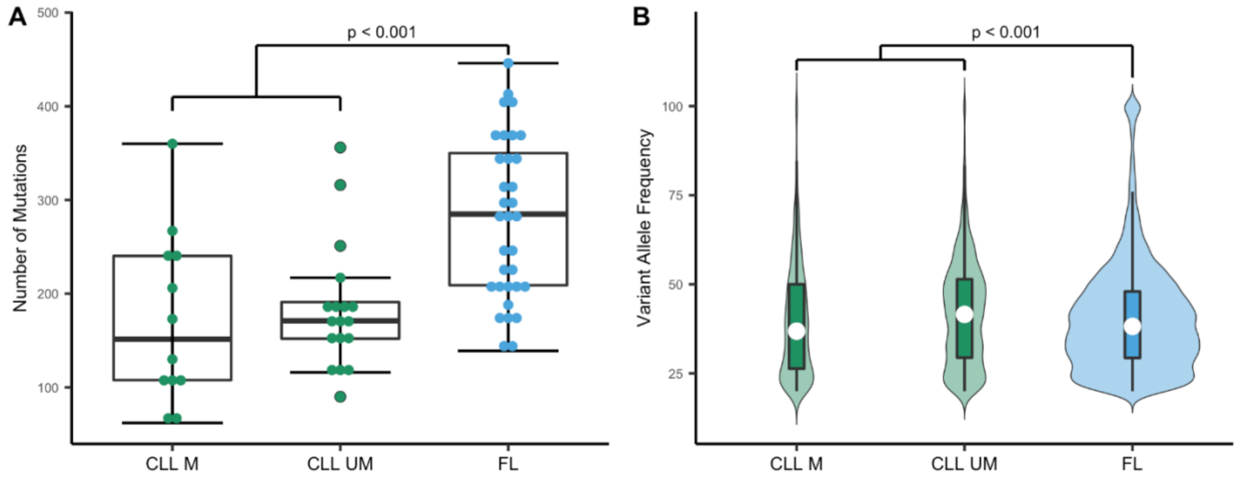

Supplementary Figure 4. 96-trinucleotide mutational pattern of FL and CLL/MBL divided on genome-wide (WES) and in immunoglobulin genes loci (IG).

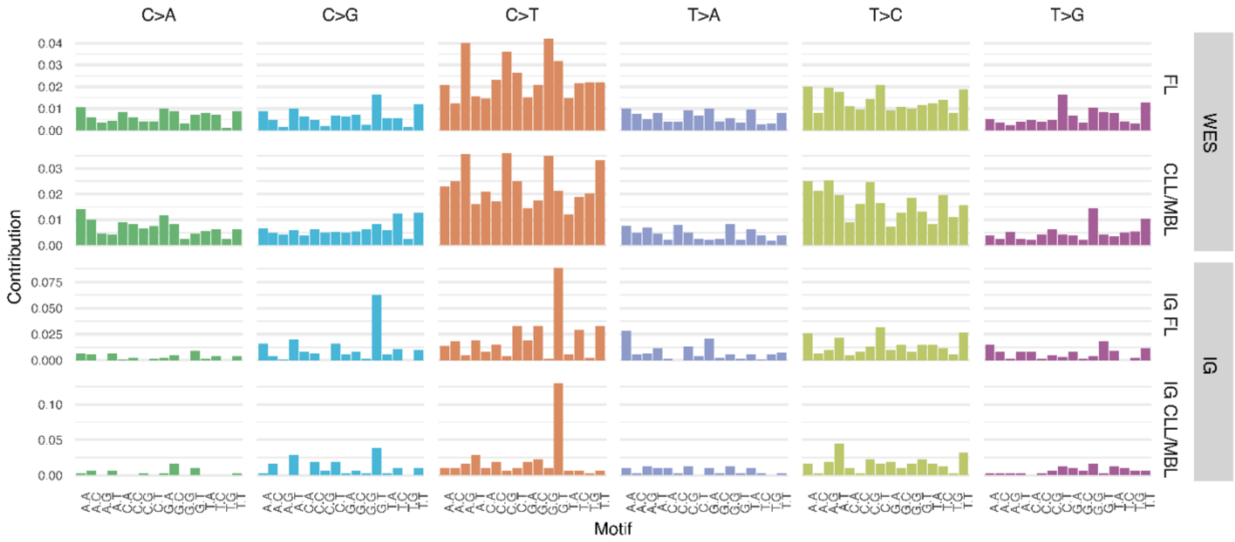

Supplementary Figure 5. Characterization of immunoglobulin loci (IG) in FL and CLL samples. **A** Cosine similarity measure between the mutational profile in immunoglobulin loci (IG) (showed in Fig. 4) and the Single Base Substitution (SBS) signatures from the COSMIC catalog (v3.2). It is shown the 15 most similar patterns in COSMIC. **B** AID motifs contribution within IG loci for each sample. (MU) indicates CLL/MBL cases with mutated IGHV.

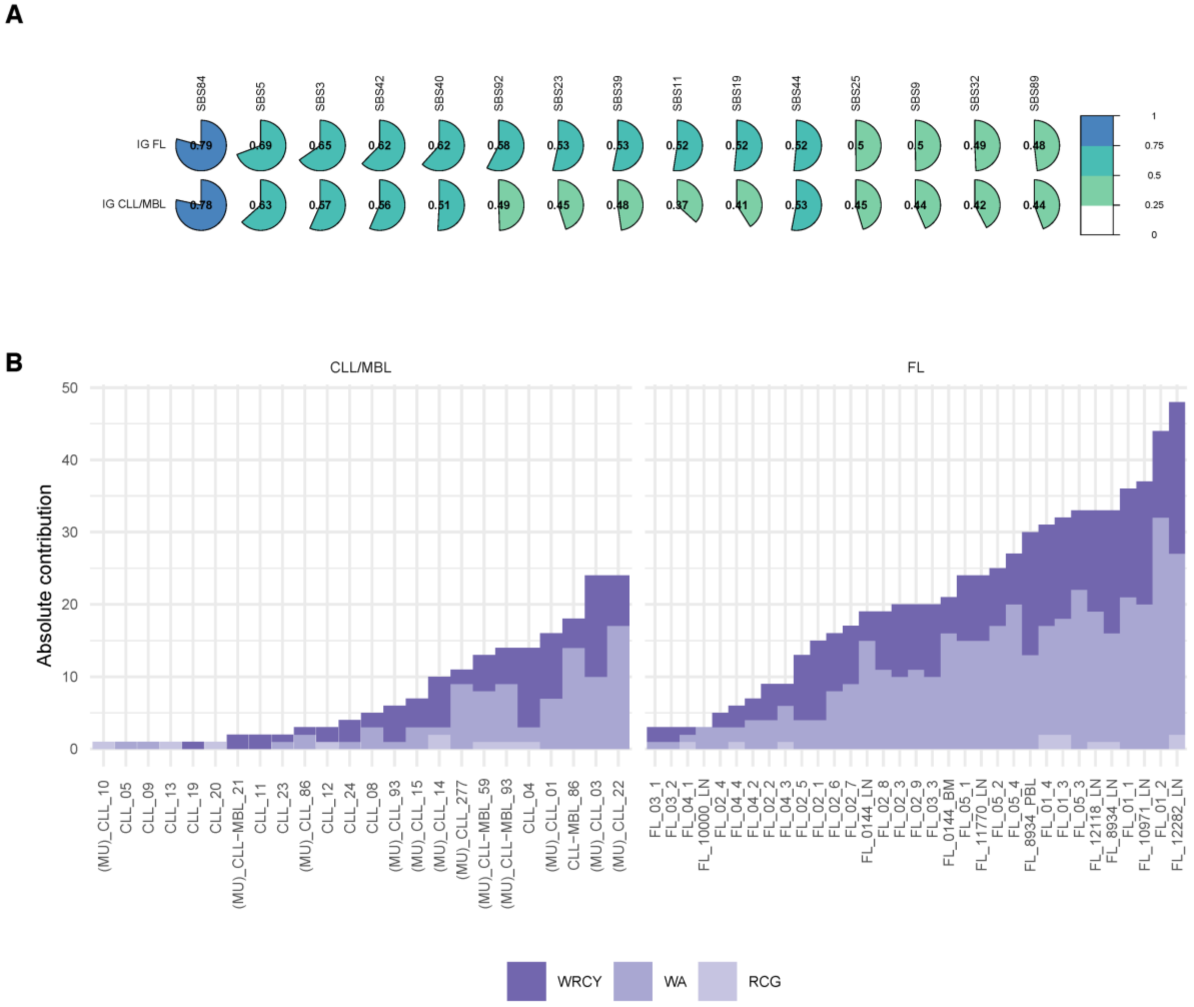

Supplementary Figure 6. Consistency of signature extraction with sample bootstrapping. To evaluate the consistency of signature extraction, NMF was performed in different sample subsets. Extraction 1: Only CLL (chronic lymphocytic leukemia) samples. Extraction 2: Only FL (follicular lymphoma) samples. Extraction 3: FL pretreatment samples. Extraction 4: FL after treatment samples. Every signature extraction (sample subset) produced two signatures (*_S1 and *_S2) and it was compared independently against the complete (63 samples) *de novo* extraction with 3 signatures (GC, SBS3+SBS6, SBS1+SBS5). Signatures are compared using cosine similarity.

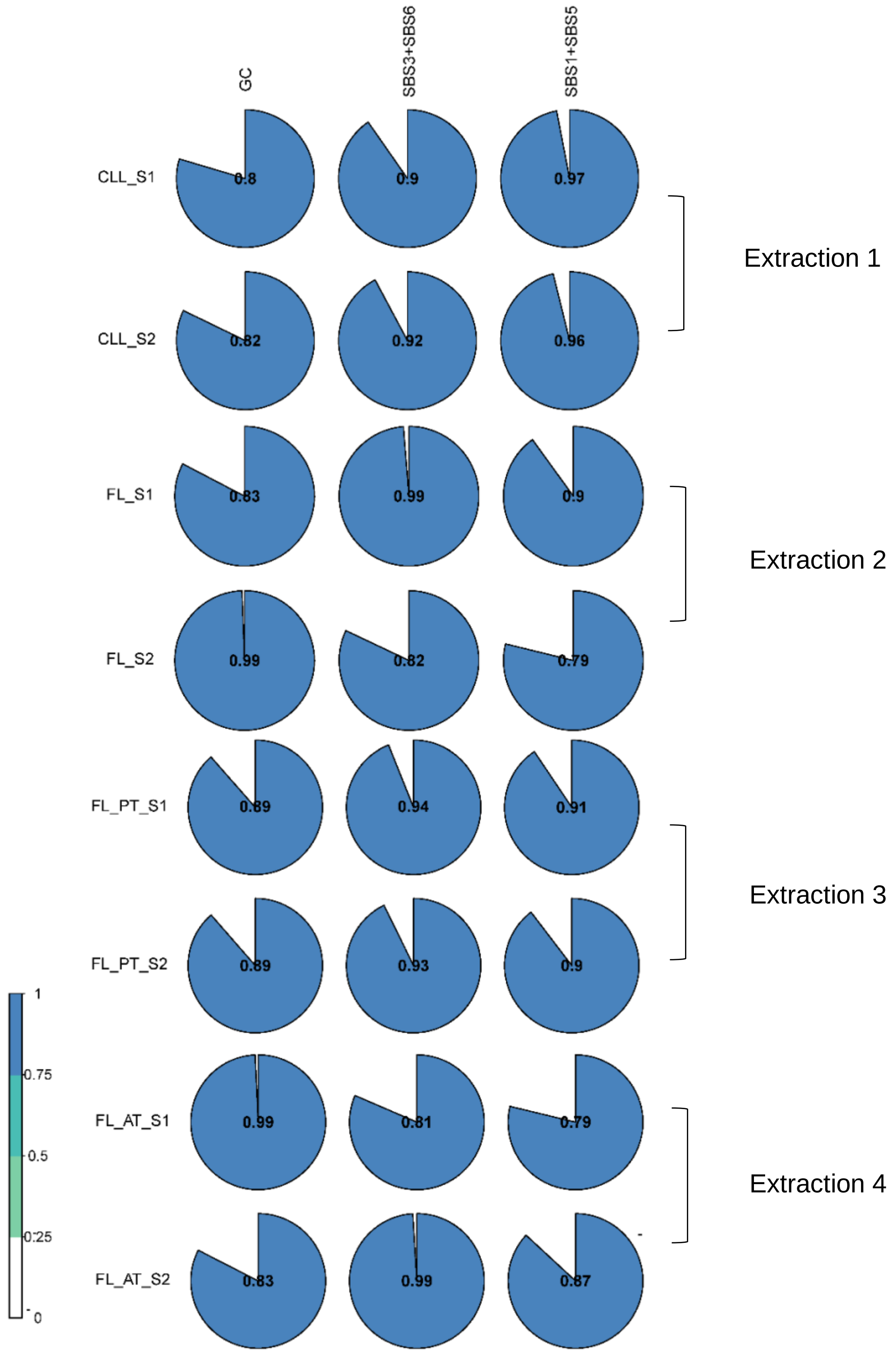

Supplementary Figure 7. *De novo* mutational signatures extraction in immunoglobulin gene loci (IG). **A** Two mutational signatures were obtained after de novo deconvolution, i.e. signature “immunoglobulin 1” (Sig. IG1), and signature “immunoglobulin 2” (Sig. IG2). **B** Prevalence of the *de novo* extracted mutational signatures to individual tumor samples. Each bar represents an individual exome. **C** Fitting using de novo and COSMIC signatures.

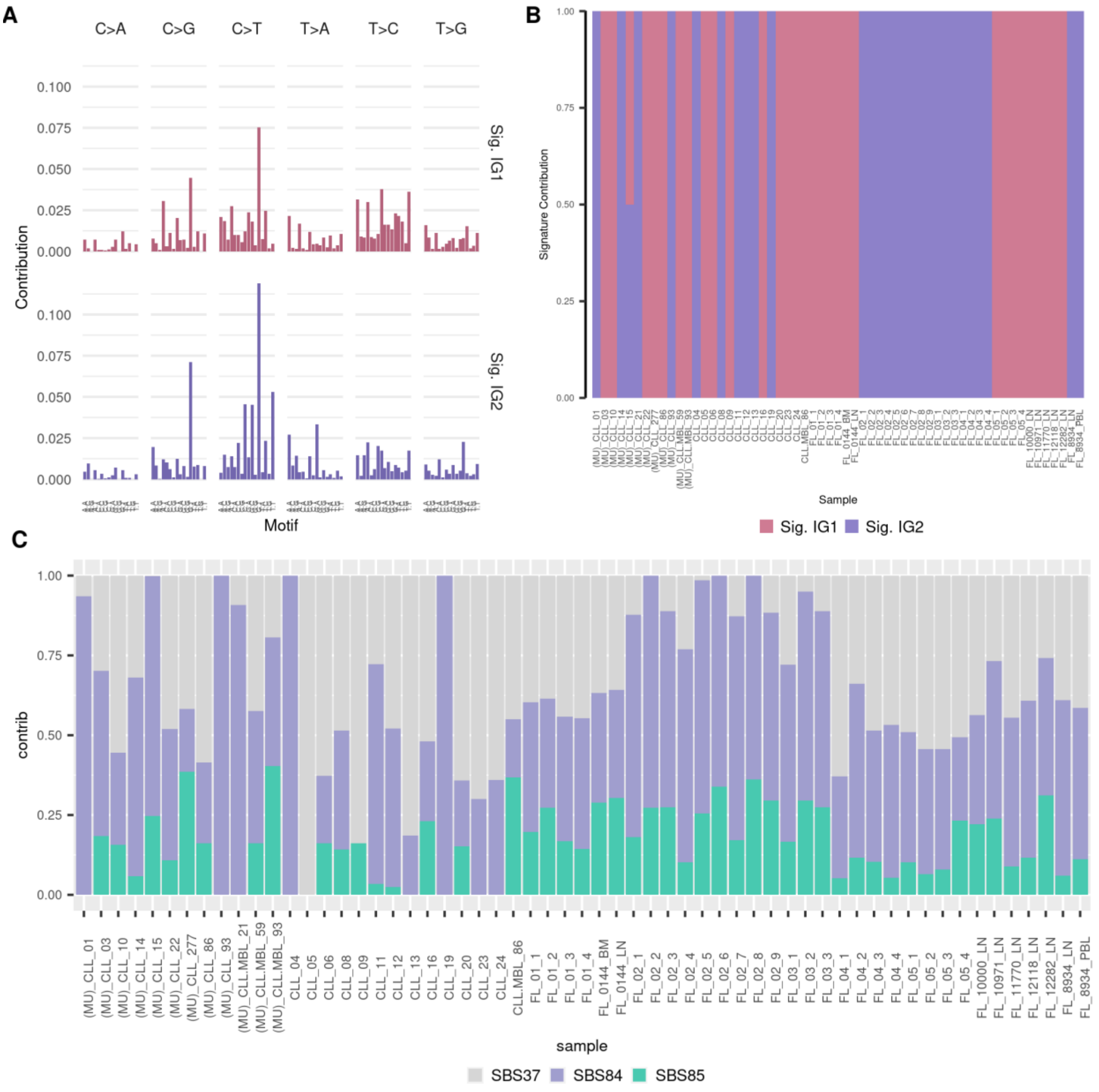

Supplementary Figure 8. Heatmap of DNA repair genes mutated in FL and CLL/MBL. The genes are divided by pathways, every column represents a defined mutation and substitution type.

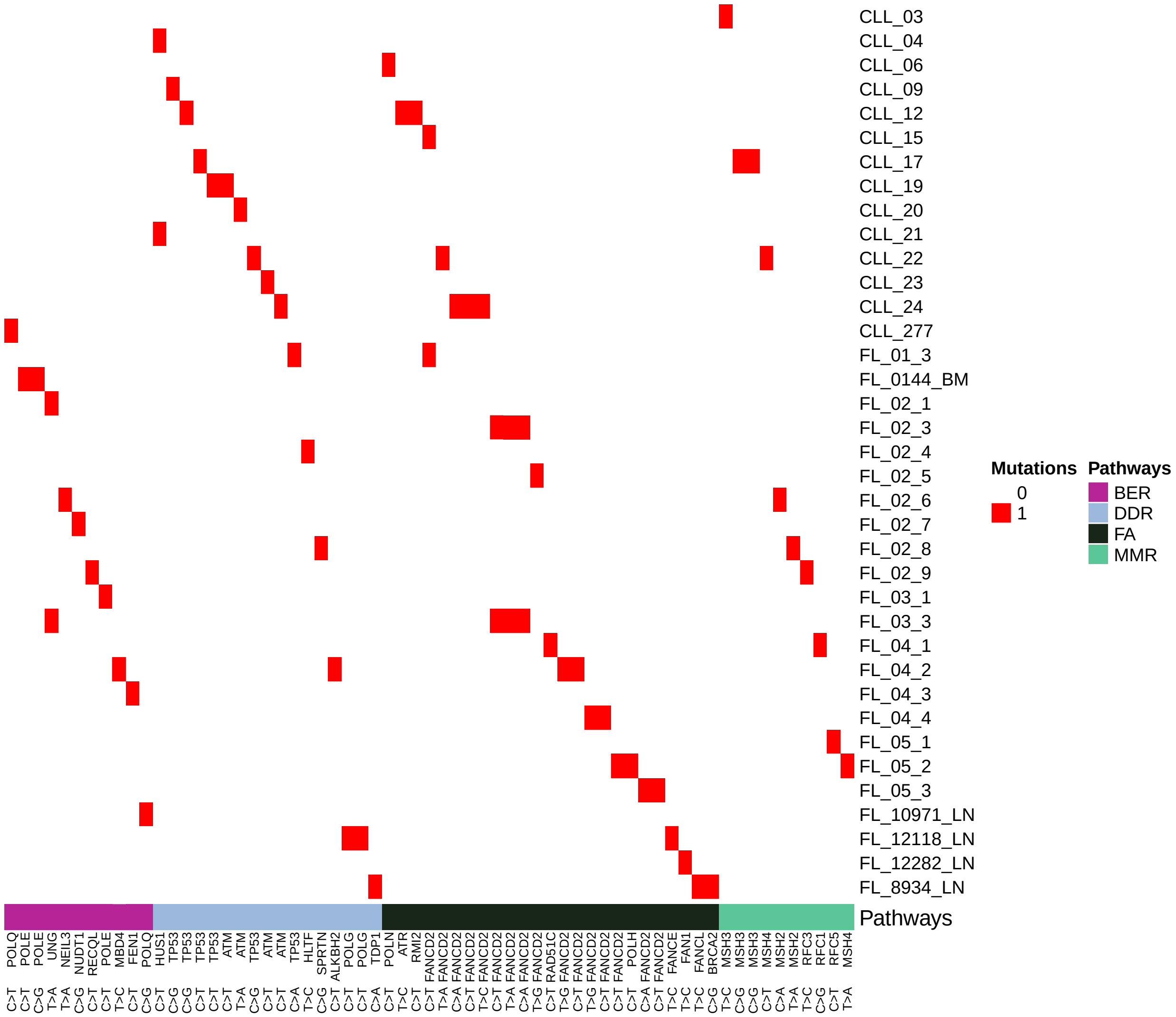

Supplement: Supplementary file 1 [file ijms-22-13015-s001.zip › Suplementary_Figures_v2_clean.pdf]
